# A multidisciplinary program of preparation for childbirth and motherhood: maternal anxiety and perinatal outcomes

**DOI:** 10.1186/1742-4755-7-28

**Published:** 2010-10-29

**Authors:** Elenice B Consonni, Iracema MP Calderon, Marcos Consonni, Marta HS De Conti, Tânia TS Prevedel, Marilza VC Rudge

**Affiliations:** 1 Department of Neurology, Psychology and Psychiatry, Botucatu School of Medicine, Univ Estadual Paulista, Botucatu, Brazil; 2 Department of Gynecology and Obstetrics, Botucatu School of Medicine, Univ Estadual Paulista, Botucatu, Brazil; 3 Departament of Health Sciences, Physiotherapy School, Universidade Sagrado Coração/USC, Bauru, Brazil; 4 Department of Health Sciences, Physical Therapy Program, São Paulo Federal University/Unifesp, Santos, Brazil

## Abstract

**Background:**

To study maternal anxiety and perinatal outcomes in pregnant women submitted to a Multidisciplinary Program for Childbirth and Motherhood Preparation (MPCM).

**Methods:**

This is a not randomized controlled trial on 67 nulliparous pregnant women divided into two groups according to participation (MPCM Group; n = 38) or not (Control Group; n = 29) in MPCM. The program consisted of 10 meetings (between the 18th and the 38th gestational week) during which educational, physiotherapeutic and interaction activities were developed. Anxiety was quantified at the beginning and at the end of the gestational period by the Trace-State Anxiety Inventory (STAI).

**Results:**

Initial maternal anxiety was equivalent between the groups. At the end of the gestational period, it was observed that anxiety levels increased in the Control Group and were maintained in the MPCM Group. A higher occurrence of vaginal deliveries (83.8%) and hospital discharge of three-day-older newborns (81.6%) as a result of MPCM was also significant. Levels of state-anxiety at the end of pregnancy showed a negative correlation with vaginal delivery, gestational age, birth weight and Apgar index at the first minute and positive correlation with the hospital period remaining of the newborns.

**Conclusion:**

In the study conditions, MPCM was associated with lower levels of maternal anxiety, a larger number of vaginal deliveries and shorter hospitalization time of newborns. It was not related to adverse perinatal outcomes.

## Background

The pregnancy experience can be seen as a major occurrence in women's global development. It is an extremely significant transition moment which causes deep physical and emotional changes, thus requiring numerous adaptations [1-4]. As much as a woman has desired to become a mother, the fulfillment of such desire may not bring the expected feelings for her new role of a pregnant woman or of a mother.

Maldonado [1] refers to pregnancy as one of the "crisis" or transition periods in a woman's normal development process, similarly to adolescence and climacterium. Any of these phases involves the need for restructuration and readjustment in various realms: biochemical, psychological, and socioeconomic, which justifies the presence of a certain level of anxiety. However, when such anxiety becomes very intense, the possibilities of obstetric complications in pregnancy, delivery and puerperium increase [1,5].

The way how emotional states interfere in pregnancy is little known. Certain conditions, such as anxiety, in addition to negatively affecting pregnancy as the mediators of endocrine alterations, may cause risk behaviors, such as delayed and/or inadequate access to prenatal care, smoking, alcohol and drug abuse, inadequate diet and gestational weight gain [6-9].

Pregnancy anxiety has been noted for negative neonatal outcomes, including a greater incidence of low birth weight and prematurity, and it has also been associated with lower Apgar scores [10-12].

In a systematic review on the scientific production on pre- and postnatal maternal anxiety from 1998 to 2003, Correia and Linhares [9] observed that the presence of high levels of maternal anxiety constitutes a potential risk factor for both maternal emotional balance and the child's development, even in the fetal period.

The participation in preparation groups during prenatal care represents an opportunity during which the psychological problems experienced in pregnancy can be coped with. Prenatal education has emerged especially to provide for the loss of the old informal support network among women themselves. Various methods of prenatal preparation have been described. Some begin in early pregnancy and mainly teach notions of hygiene and self-care; others take place in the in the last trimester and basically prepare the pregnant woman for delivery; others still concentrate on the motherhood experience [13].

Although delivery preparation methods have existed from the early 20th century, the literature lacks studies showing the action of such methods on maternal anxiety rates and obstetric complications. Interventions are performed during pregnancy and/or delivery and range from educational programs, the use of physical and mental relaxation techniques, coping strategies, psychosocial advice and hypnosis [14-18].

In the last few decades, the literature has been enhanced by an increasing number of studies whose results show the association between psychological factors in pregnancy and their outcomes in gestation, delivery, puerperium and even in the newborn's later development [9,11,19-21]. These findings consider the possibility of predicting complications in pregnancy through the application of psychological tests during the prenatal period as well as of reducing such occurrences by instituting preparation programs for delivery and motherhood [5,9,11].

This research aimed to study maternal anxiety and perinatal outcomes in pregnant women submitted to a Multidisciplinary Program for Childbirth and Motherhood Preparation (MPCM).

## Methods

This is a not randomized controlled trial on 67 nulliparous pregnant women divided into two groups according to participation (MPCM Group; n = 38) or not (Control Group; n = 29) in MPCM.

Pregnant women followed during low-risk prenatal care at the Botucatu School of Medicine (FMB/UNESP), which assists primigravid women and adolescents, participated in the study.

A possible sample was used; hence, sample size was determined by the flow of primigravid women (eight new cases/month) and by the project's development schedule (12 months/48 weeks for subjects' inclusion), which would allow for including at the most 96 primigravid women. Considering the proportion of 20% of pregnant women who are referred for high-risk care and who would consequently not be eligible for inclusion in the study, the possible sample resulted in 80 pregnant women, distributed into the two groups.

Eligibility criteria were: nulliparity; absence of clinical or obstetric diseases, single pregnancy, gestational age between the 18^th ^and 22^nd ^weeks and agreement to participate in a childbirth preparation program. Regardless of which group they were assigned to, all the pregnant women signed a Free Consent Form, thus confirming their agreement to participate in the study.

Discontinuity criteria were prenatal care interruption, delivery out of the service's facilities and non-adherence to MPCM, which was defined by the absence from two consecutive or non-consecutive meetings.

MPCM consisted of 10 meetings, the first six of which were held fortnightly while the last four occurred weekly. Each meeting lasted three hours and comprised three basic activities: educational, physiotherapeutic and interaction. The meetings began with the educational activity, with an approximate duration of 50 minutes. After an interval of 15 to 20 minutes, during which a snack was offered, half of the pregnant women alternately participated in the other activities, namely physiotherapeutic and interaction, for an average period of 50 minutes each. The intervention program was developed and applied by a multidisciplinary team, and each of the three activities was supervised by qualified professionals from the three areas.

The educational activity provided information concerning pregnancy, delivery, puerperium and care for the newborn. The previously selected topics were presented by health care professionals and discussed with the pregnant women rather informally. A visit to the maternity unit was included, and the pregnant women visited the obstetric ward, the shared lodging, prepartum and delivery facilities and the neonatal unit. During the physiotherapeutic activity, resources including respiratory training, postural orientation for activities of daily living, kinesiotherapy practices (stretching and muscle strengthening) and relaxation techniques were applied. After the 36^th ^week, the pregnant women received orientation and training on breathing techniques and maneuvers for the expulsion period of delivery. The interaction activity represented an opportunity for discussing the pregnancy experiences, the emotional experiences involved in the situation of having a child and the impact of pregnancy in the family context. The main topic of the discussion was the subject presented by the health care professionals in the educational activity although it could be diverted according to the group's interest at that moment. The last fifteen minutes were reserved for physical and mental relaxation.

The independent variable was participation or not in MPCM. The control variables were defined by: maternal age, marital status, schooling; current activity (study and work activities) and social support (reference to any type of emotional, financial and/or practical support within or out of the family circle).

The following were analyzed as dependent variables: levels of maternal anxiety, delivery route (categorized as vaginal and caesarean); gestational age at birth (classified as < 37 and ≥ 37 weeks); the newborn's weight (categorized as < 2500 and ≥ 2500 g); Apgar indexes (at the 1st and 5th minutes of life, considering < 7 and ≥ 7) and the newborn's hospitalization period (classified as ≤ 3 and > 3 days).

The State-Trait Anxiety Inventory (STAI), designed and standardized by Spielberg et al. [22], was used to evaluate anxiety. This instrument differentiates and evaluates two anxiety types: anxiety as a trait - Trait Anxiety - and state or situational anxiety - State Anxiety. The anxiety trait refers to individual differences concerning the tendency to respond to situations perceived as threatening, that is, stable and relatively permanent personality characteristics. The anxiety state is considered to be a transitory emotional state or an organism's temporary condition that is characterized by consciously perceived unpleasant feelings of tension and apprehension and by increased activity of the autonomic nervous system. STAI has been frequently used in the field of research and provides an adequate measurement of anxiety levels in pregnant women [23].

Maternal anxiety was evaluated at two gestational moments: between the 18th and the 22nd weeks, when the complete inventory was applied with two anxiety scales - Trait Anxiety (TA) and State Anxiety (SA1), and between the 36th and the 38th weeks, when only the State Anxiety scale (SA2) was used. The level of anxiety was quantified by the scores obtained on each pregnant woman's scale.

The per-protocol (PP) analysis was defined as the mode for result analysis. For statistical analysis, the χ2 test was used with Yates' correction for comparison of proportions (frequencies). In order to study anxiety characteristics between and within groups, the t test for independent and dependent samples was used. The simple linear correlation coefficient (r) was utilized to analyze the possible correlations between anxiety characteristics and perinatal outcomes. For all statistical tests, 5% was adopted as the limit of significance (*p *< 0.05).

This project was previously approved by the Research Ethics Committee of the Botucatu School of - Unesp.

## Results

**Figure 1:**
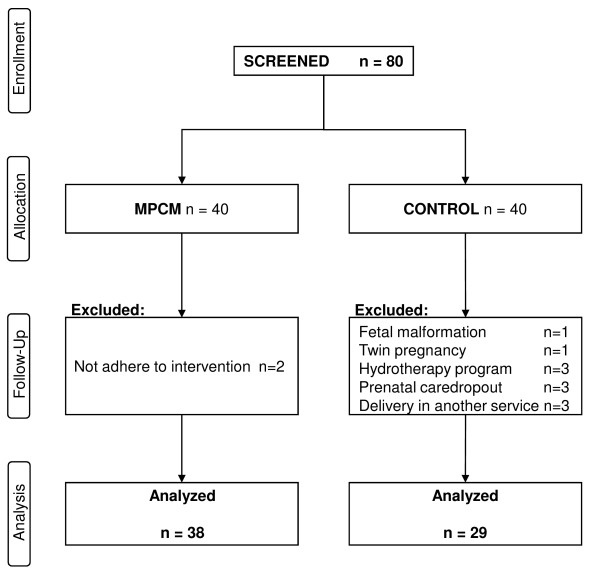
**Flow chart of the subjects through the phases of the study**.

Figure 1 shows the flow chart with details on the classification, allocation in the groups, follow-up and evaluation of the studied subjects. Eighty pregnant women were included, and they were equally distributed in the Control (N = 40) and MPCM (N = 40) Groups. Two pregnant women were excluded from MPCMG due to non-adherence to MPCM, that is, they were absent from two or more meetings. Of the forty women in CG, 11 were not considered in the analysis of results: two due a late diagnosis of gemelarity and fetal malformation, three due to voluntary adherence to a hydrotherapy program, three due to the fact that their delivery occurred out of the service's facilities and three others for interrupting prenatal care in the service. Hence, the results of 38 women in MPCMG and of 29 in CG were compared.

No significant differences were found between the MPCMG and CG as regards the sociodemographic variables (Table 1). Regardless of groups, approximately 60% of the pregnant women were younger than 18 years old. Most of them reported to have a steady partner and had attended secondary school. One fourth of the women were still attending school. In MPCMG, 15.8% of the women worked, and in the Control Group, such percentage was of 27.6%, but that difference was not significant. In the two groups evaluated, the majority of the pregnant women reported to have some type of emotional, financial and/or practical support within or out of their family circles (Table 1).

**Table 1:** Maternal characteristics

	MPCM GROUP		CONTROL GROUP	
	n	%	n	%
Age (years)*				
≤ 18 years	23	60.5	16	55.2
> 18 years	15	39.5	13	44.8

Marital status*				
Lives with a partner				
yes	26	68.4	25	86.2
not	5	13.2	3	10.3
Do not have a partner	7	18.4	1	3.5

Schooling*				
Elementary school	17	44.7	13	44.8
High school	19	50.0	15	51.7
College	2	5.3	1	3.5

Current occupation*				
Student	10	26.3	8	27.6
Worker	6	15.8	8	27.6

With social support*	31	81,6	25	86,2

* p > 0.05 - non-significant difference by the χ2 test.

The Trait-Anxiety (T) and State-Anxiety (S) means for the studied groups are shown in Table 2. No statistical difference was observed between the groups when Trait and State Anxiety were analyzed at the beginning of pregnancy (S1). State Anxiety as evaluated at the end of pregnancy (S2) was lower in the group participating in MPCM.

**Table 2:** Maternal anxiety and perinatal outcome

	MPCM GROUP		CONTROL GROUP	
	**m**	**sd**	**m**	**sd**

Maternal Anxiety				
T (Trait)	44.2	10.1	41.9	9.8
S1 (initial State)	39.3	8.6	41.1	9.6
S2* (final State)	39.1	8.2	44.2	9.9

	n	%	n	%

Gestational age at birth				
< 37 weeks	1	2.6	2	6.9
≥ 37 weeks	37	97.4	27	93.1

Delivery**				
Vaginal	31	81.6	17	58.6
Caesarean	7	18.4	12	41.4

Weight				
< 2,500 g	3	7.9	4	13.8
≥ 2,500 g	35	92.1	25	86.2

Apgar 1^st^				
< 7	3	7.9	4	13.8
≥ 7	35	92.1	25	86.2

Apgar 5^th^				
< 7	0	-	0	-
≥ 7	38	100	29	100

Hospitalization time*				
≤ 3 days	31	81.6	13	44.8
> 3 days	7	18.4	16	55.2

* p < 0.05 - significant difference by the independent t test.

** p < 0.05 - significant difference by the χ^2 ^test.

The occurrence of vaginal delivery predominated in the MPCMG (81.6%) and, contrarily, the incidence of caesarian delivery was higher in the CG (41.4%). Such differences were statistically significant (Table 2).

Despite the non-significant difference, a lower frequency of preterm (2.6% *vs*. 6.9%) and low-weight (7.9% *vs*. 13.8%) newborns as well as of newborns with first-minute Apgar < 7 (7.9% *vs*. 13.8%) were observed in the MPCMG. All the newborns, regardless of their groups, showed Apgar ≥ 7 at the fifth minute of life. Most of the newborns whose mothers participated in MPCM (81.6%) were discharged from hospital until their third day of life, and this result was statistically different from that in CG (44.8%). In this group, more than half of the newborns (55.2%) were only discharged after the 3rd day of life (Table 2).

The levels of State-Anxiety at the end of pregnancy (S2) were inversely correlated to the frequency of vaginal delivery, gestational age at birth, the newborn's weight and Apgar indexes at the first minute of life. Such results also showed direct relation to the newborns' hospitalization time, and all the correlations were statistically significant. Initial State-Anxiety (S1) showed inverse and significant relation with the first-minute Apgar indexes (Table 3).

**Table 3:** Coefficient of correlation (r) between maternal anxiety characteristics - Trait (T), Initial State (S1) and Final State (S2), and perinatal outcomes.

	ANXIETY		
	T	S1	S2
Vaginal delivery	0.12	- 0.01	- 0.27 *
Gestational age	0.01	0.15	- 0.25 *
Weight	- 0.02	- 0.06	- 0.38 *
Apgar - 1st minute	- 0.23	- 0.28 *	- 0.30 *
Hospitalization time	0.28 *	0.21	0.36 *

* p < 0.05 - significant correlation by simple regression analysis.

## Discussion

The results in this study showed that the MPCM intervention was associated with lower State-Anxiety levels at the end of pregnancy (S2) and with better results for delivery mode and neonatal morbidity. The final anxiety (S2) levels showed an inverse relation to the occurrence of vaginal delivery, weight at birth and Apgar indexes at the first minute, and they were directly related to the newborns' time of stay in hospital. Regardless of the limitations, these results show the validity of childbirth preparation programs.

The lack of physical space, material and personnel prevented a random selection (randomization), and five MPCMG subgroups were formed each one consisting of 6 to 8 pregnant women) subsequently, until 40 participants were gathered. The CG participants were selected after each MPCMG sub-group was completed, and recruitment also occurred until 40 pregnant women were grouped.

In the two groups, Control and MPCM, most of the pregnant women were adolescents at a minimum age of 18 years. In Brazil, the problem related to teenage pregnancy is reported as social and public-health issue. In 2007, there were 2,795,207 births in the country, of which 594,205 (21.3%) were from mothers aged 10 to 19 years [24]. Adolescent pregnancy has been associated with an increased incidence of several adverse outcomes such as low birth weight (LBW), preterm delivery and small-for-gestational-age (SGA) infants [25,26]. In addition to maternal age, studies point out unfavorable psychosocial and care conditions that can explain part of the occurrences [27]. The published literature suggests that prenatal care regimens which provide social and behavioral services along with medical care could improve both the health of the mother and the outcome of her pregnancy [28].

The risk posed by pregnancy in adolescence must be even higher when it is associated to the condition of a first pregnancy. According to the literature, tension and anxiety increase in primigravid women because they face the "fear of the unknown" [29]. In this study, despite the association of adolescence and nulliparity, this did not occur, and the correlation index value showed that the control of State-Anxiety (S2) final levels would explain at least some of the observed outcomes.

MPCM fostered anxiety control at the end of pregnancy, and this must have contributed to the newborns' favorable prognosis in this group. In the reviewed literature, no studies were found which related this type of intervention to maternal anxiety levels. This fact hindered the discussion of the results herein observed, but pointed out the singularity of the study.

The effects of preparation programs for childbirth and motherhood on perinatal outcomes are controversial among authors [30]. The differences in relation to the start moment, intervention time and the evaluated outcomes associated with the lack of controlled clinical trials and with adequate samples sizes add to such controversy.

In this scenario, the results in this study must be interpreted with limitations. The characteristics of the program developed, including differentiated group activities, and the restrictions from the physical space and time for developing the project made randomization and the inclusion of a larger number of subjects unfeasible. Discontinuity by more than 20% of the pregnant women in the control group, most of whom were at the beginning of the intervention, did not enable analysis by means of treatment intention, and that was a limiting factor for result interpretation. On the other hand, similar maternal characteristics in the two groups and the statistically significant results, even with a small sample size, contribute to minimize such limitations.

The beneficial potential, the absence of risk for newborns and above all, the possibility of humanized care [29] justify the continuity of studies aiming at defining the results of this intervention type.

## Conclusions

In the study conditions, MPCM was associated with lower levels of maternal anxiety, a larger number of vaginal deliveries and shorter hospitalization time of newborns. It was not related to adverse perinatal outcomes.

## List of abbreviations

MPCM: Multidisciplinary Program for Childbirth and Motherhood; MPCMG: MPCM Group; CG: Control Group; FMB: Botucatu School of Medicine; STAI: State-Trait Anxiety Inventory; T: Trait-Anxiety; S1: Initial State-Anxiety; S2: Final State-Anxiety.

## Competing interests

The authors declare that they have no competing interests.

## Authors' contributions

EBC, IMPC and MHSC participated in all steps of the study, including research and program planning, data collection, analysis and manuscript writing. MC, TTSP and MVCR participated in the program planning as well as in manuscript analysis and review. All authors have read the manuscript and agreed on its content.
